# Increasingly transformed MCF-10A cells have a progressively tumor-like phenotype in three-dimensional basement membrane culture

**DOI:** 10.1186/1475-2867-9-7

**Published:** 2009-03-16

**Authors:** Karen M Imbalzano, Iva Tatarkova, Anthony N Imbalzano, Jeffrey A Nickerson

**Affiliations:** 1 Department of Cell Biology, University of Massachusetts Medical School, Worcester, MA 01655, USA

## Abstract

**Background:**

MCF-10A cells are near diploid and normal human mammary epithelial cells. In three-dimensional reconstituted basement membrane culture, they undergo a well-defined program of proliferation, differentiation, and growth arrest, forming acinar structures that recapitulate many aspects of mammary architecture *in vivo*. The pre-malignant MCF-10AT cells and malignant MCF-10CA1a lines were sequentially derived from the MCF-10A parental cell line first by expression of a constitutively active T24 H-Ras generating the MCF-10AT cell line. This was followed by repeated selection for increasingly aggressive tumor formation from cells recovered from xenograft tumors in immuno-compromised mice, generating the MCF-10CA1a cell line. When inoculated subcutaneously into the flanks of immuno-compromised mice, MCF-10AT cells occasionally form tumors, whereas MCF-10CA1a cells invariably form tumors with a shorter latency than MCF-10AT derived tumors.

**Results:**

MCF-10AT cells grown in three-dimensional basement membrane culture form complex multi-acinar structures that produce a basement membrane but undergo delayed cell cycle arrest and have incomplete luminal development. MCF-10CA1a cells grown in three-dimensional basement membrane culture form large, hyper-proliferative masses, that retain few characteristics of MCF10A acini and more closely resemble tumors.

**Conclusion:**

Here we report on the growth and differentiation properties of these three matched cell lines in three-dimensional basement membrane culture. Features of tissue morphogenesis were assessed, including proliferation, basement membrane formation, polarization of alpha-6 beta-4 integrin to the basement membrane, formation of cell:cell junctions, and apoptosis for luminal clearance. The matched series of normal MCF-10A, pre-malignant MCF-10AT, and malignant MCF-10CA1a cells offers a unique opportunity to study the mechanisms of malignant progression both in a three-dimensional microenvironment and in the same cell background.

## Background

Universal features of breast cancer include a loss of control of cell proliferation and organization, a loss of cell polarity, as well as a loss of cell: cell adhesion and cell: basement membrane adhesion. Monolayer cultures of mammary epithelial cells do not closely mimic these features of *in vivo *cell and tissue architecture [1,2], and also do not recapitulate the alterations in nuclear structure characteristic of breast tumors [3]. The development of three-dimensional culture systems for mammary epithelial cells has been an important advance in cell culture models, more closely mimicking *in vivo *architecture and are, therefore, more relevant systems in which to elucidate changes in cell growth, cell: cell adhesion, cell: cell junctions, and cell: extra-cellular matrix (ECM) interactions that occur during malignant progression [1,4,5].

MCF-10 cells were derived from a patient with fibrocystic disease and the immortalized MCF-10A line arose spontaneously in culture [6]. MCF-10 cells are diploid, while the MCF-10A line has a stable, near-diploid karyotype [6,7] with modest genetic modifications typical of culture-adapted breast epithelial cells [8] including loss of the p16 locus [9]. The cells express normal p53 [9,10], they do not form colonies in soft agar, and they do not grow in immuno-compromised mice [11].

Several transformed lines have been derived from MCF-10A cells. The MCF-10AT line was created by forced expression of activated H-ras [12,13]. Implanted as xenografts, MCF-10AT cells formed nodules that progressed from hyperplasia to carcinoma *in situ *in 25% of mice [12,14]. Further selection for tumor formation in mice generated the series of MCF-10CA1 cell lines that are fully malignant, producing tumors in 100% of immuno-compromised mice transplanted with the cells [15,16]. Thus, the MCF-10A series of matched cell lines provides a complete spectrum of cell phenotypes, from normal to pre-malignant to fully malignant.

MCF-10A cells cultured in three-dimensional reconstituted basement membrane culture (rBM) develop important features of normal breast tissue via a well described progression of proliferation, cell cycle arrest, apical-basolateral polarization, and finally, apoptosis to create a luminal space [9,17,18]. In addition, cell nuclei of MCF-10A cells forming acini in three-dimensional rBM culture display a reorganized and differentiated nuclear architecture more characteristic of mammary epithelial cells in tissue than those cultured in monolayer [3]. The resulting acini of MCF-10A three-dimensional rBM cultures resemble those of normal breast tissue, which are clustered in lobules that connect to intralobular ductules that, in turn, connect to interlobular ducts.

The MCF10A series of cell lines provides a unique opportunity to probe malignant progression induced in a molecularly defined way, in a common cell background. We have characterized their ability to form tissue-like structures in a three-dimensional microenvironment. In contrast to MCF-10A cells, MCF-10ATcells, when cultured in three-dimensional in rBM, form multi-acinar structures, and MCF-10CA1a cells, when also cultured in three-dimensional in rBM, form large hyper-proliferative cell aggregates with altered organization and no lumen. We conclude that pre-malignant cells have lost normal proliferation control but retain normal cell polarity, cell:cell, and cell:basement membrane adhesion. Fully malignant cells, on the other hand, have lost the ability to undergo normal acinar morphogenesis. The application of three-dimensional rBM culture to this unique progression series of cell lines with a matched cell background provides a physiologically relevant *in vitro *model of breast cancer progression.

## Results

### Monolayer cultures

**Figure 1:**
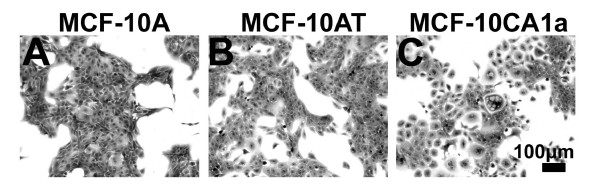
**Cell colony morphology observed for MCF-10A, MCF-10AT, and MCF-10CA1a cells grown in monolayer**. Phase contrast images of normal MCF-10A (A), transformed MCF-10AT (B) and malignant MCF-10CA1a (C) grown in monolayer. Scale bar, 100 μm.

In monolayer culture, MCF-10A and MCF-10AT cells grew in expanding colonies with the cobblestone appearance characteristic of epithelial cells. (Figure 1A, B) MCF-10CA1a cells had a less cobblestone-like appearance, occasionally had a more spindle-like shape and fibroblastic appearance, and often had cytoplasmic vacuoles (Figure 1C). This is consistent with previous reports of morphological changes and a more mesenchymal appearance for MCF-10CA1a cells [15].

### Multi-acinar and hyper-proliferative mass formation in three-dimensional culture by MCF-10AT and MCF-10CA1a cells respectively

**Figure 2:**
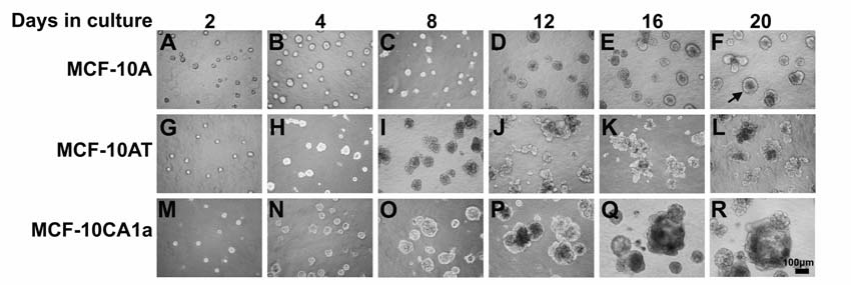
**Morphology observed for MCF-10A, MCF-10AT, and MCF-10CA1a cells grown in rBM for 20 days**. Phase contrast images of normal MCF-10A cells were cultured in overlay rBM for 2, 4, 8, 12, 16, and 20 days (top, A to F). Transformed MCF-10AT cells (middle, G to L) and malignant MCF-10CA1a cells (bottom, M to R) were cultured in three-dimensions at the same time points. Scale bar, 100 μm. Normal MCF-10A cells increase in size to day 8 and form polarized spherical acini. Transformed MCF-10AT cells increase in size for 20 days, retain polarity, and form multi-acinar structures. Malignant MCF-10CA1a cells continue to proliferate throughout the 20 days and form large, complex masses.

Parental MCF-10A, pre-malignant MCF-10AT, and malignant MCF-10CA1a cell lines were cultured in rBM. In this procedure, cells were plated on a layer of rBM with an overlay created by adding rBM to the culture medium [9]. After two days, cultures of all three cell lines formed similarly-sized spherical masses of cells (Figure 2A, G, M). After 4 days, the masses of MCF-10AT and MCF-10CA1a cells were larger than the masses of MCF-10A cells (Figure 2B, H, N), an indicator of increased proliferation. After 8 days in three-dimensional culture, the MCF-10AT structures had an acinar appearance but these acini were larger than those for the parental MCF-10A cells and were more irregular in shape (Figure 2C and 2I).

Between days 8 and 12 in three-dimensional culture, the MCF-10A acini continued to increase in size while retaining their overall spherical shape (Figure 2C, D). During this same time, MCF-10AT acini not only continued to increase in size, but also in many cases appeared to have generated a multi-acinar structure (Figure 2I, J). Although some of the MCF-10CA1a structures at day 12 remained the same size as on day 8 they often appeared darker and denser. Other MCF-10CA1a structures appeared to be masses of disorganized cells that continued to increase in size between days 8 to day 12 (Figure 2O, P).

**Figure 3:**
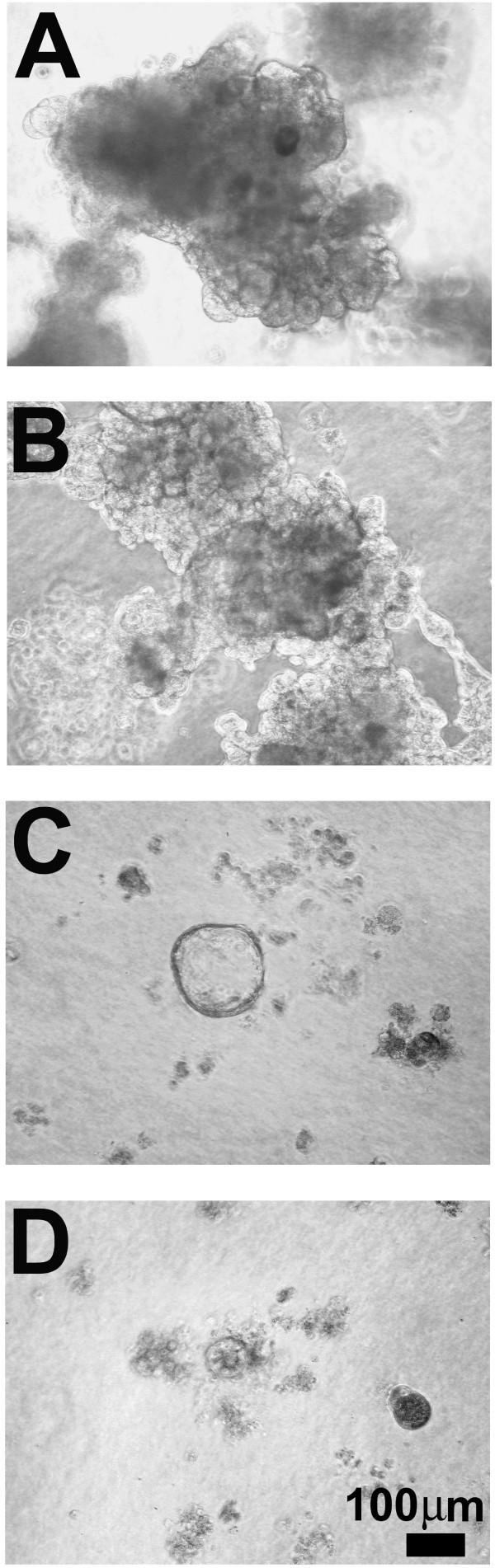
**Range of morphologies observed for MCF-10CA1a cells grown in rBM for 20 days**. Phase contrast micrographs of malignant MCF-10CA1a cells after 20 days in overlay three-dimensional culture which form large defined dense masses (A), more spread, less defined masses (B), highly vacuolar acini (C), and clusters of MCF-10CA1a cells that have failed to thrive in rBM (D). Scale bar, 100 μm.

From day 12 to day 20 in three-dimensional culture, the MCF-10A acini did not appreciably change in size or shape, consistent with a cessation of proliferation [4,9]. Acini remained spherical with a smooth outer edge and when the peripheral edge of the acinus was in the focal plane, individual cells at that edge appeared of uniform size and were evenly spaced (arrow panel F). A few acini were no longer spatially separated, but were touching by these later time points (Figure 2E, F). They did not, however, appear to be fused into a single structure. The multi-acinar MCF-10AT structures continued to increase in size from day 16 to day 20 (Figure 2K, L). By days 16 to 20, MCF-10CA1a structures were larger and even more easily distinguishable from those of the MCF-10A and MCF-10AT (Figure 2Q, R). Since the growth of MCF10CA1a cells in three-dimensional culture was variable, a spectrum of typical morphologies observed at 20 days in three-dimensional culture is presented in Figure 3. Some masses of MCF-10CA1a cells were large and very dense with uneven edges (Figure 3A); whereas other masses lacked distinct edges, were less dense, and were more spread (Figure 3B). Some structures were very large with round, smooth outer edges and a vacuolar interior (Figure 3C). We also continued to see both masses of cells that did not appear organized as well as remnants of cells that failed to thrive in three-dimensional culture (Figure 3D).

**Figure 4:**
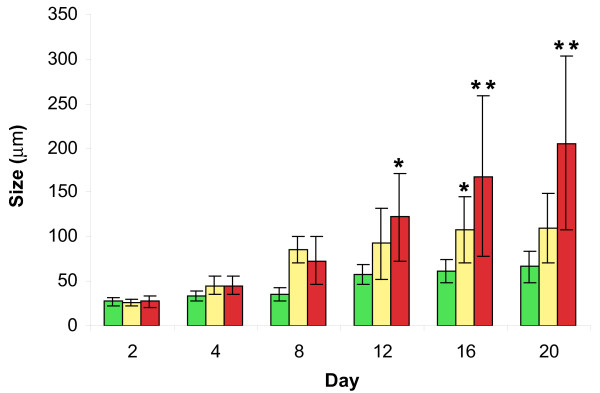
**Size distributions observed for MCF-10A, MCF-10AT, and MCF-10CA1a cells grown in rBM for 20 days**. The significance of size difference between MCF-10A (green), MCF-10AT (yellow), and MCF-10CA1a (red) cells grown in overlay rBM culture was determined by analysis of variance followed by a Tukey t-test. Data are presented as means ± SEM (n = 10). Asterisks depict statistically significant differences between groups shown (*p < 0.05, ** p < 0.001).

Quantification of the sizes of structures formed by the three cell lines in three-dimensional culture is presented in Figure 4. The size of MCF-10A acini stabilized after 12 days in three-dimensional culture, consistent with an end to proliferation at this time. At 20 days in three-dimensional culture MCF-10A acini had a mean diameter of 66.4 ± 17.7 μm. In contrast, the size of MCF-10AT multi-acinar structures stabilized after 16 days in three-dimensional culture, suggesting a delay of the end of proliferation in this cell line in three-dimensional culture. At 20 days in three-dimensional culture the MCF-10AT multi-acinar structures had a mean diameter of 109.7 ± 39.2 μm. The MCF-10CA1a masses continued to increase in size for the entire 20 day period, indicating a loss of proliferation control in this cell line. MCF-10CA1a masses averaged 205.4 ± 97.5 μm at 20 days in three-dimensional culture. The size of the structures resulting from growth in rBM, were not significantly different among the three cells lines from day 2 to day 8 in three-dimensional culture. However, by day 12 and continuing until day 20, the MCF-10A acini were significantly smaller than the MCF-10CA1a masses as determined by an ANOVA test followed by a Tukey adjustment (Figure 4). At day 16 the MCF-10A acini were also significantly smaller than the MCF-10AT multi-acinar structures (Figure 4).

In summary, MCF-10AT cells grown on rBM formed multi-acinar structures. MCF-10CA1a cells grown in rBM developed into hyper-proliferative cellular masses that did not resemble acini. Thus, the malignant MCF-10CA1a cells grown in three-dimensional culture showed increased proliferation and decreased organization compared to both normal MCF-10A and pre-malignant MCF-10AT cells.

### Lack of programmed cell death in MCF-10AT and MCF-10CA1a cells in three-dimensional cultures

**Figure 5:**
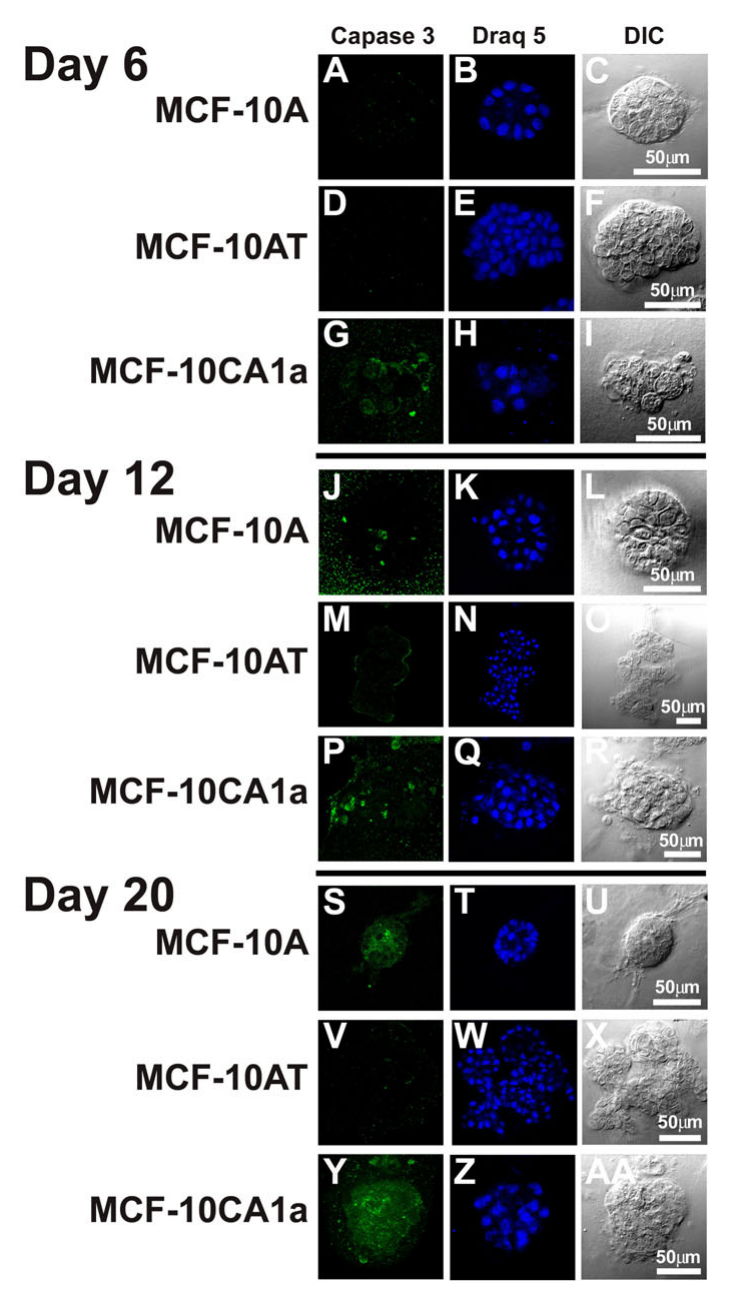
**Apoptosis observed for MCF-10A, MCF-10AT and MCF-10CA1a cells grown in three-dimensional culture**. Single confocal sections of activated caspase-3 staining (left) and nuclear staining with Draq 5 (middle) with matching differential interference contrast images (DIC-right) of MCF-10A acini in overlay three-dimensional culture at day 6 (A-C), day 12 (J-L) and day 20 (S-U); MCF-10AT multi-acinar structures at day 6 (D-F), day 12 (M-O), and day 20 (V-X); and MCF-10CA1a structures at day 6 (G-I), day 12 (P-R), and day 20 (Y-AA). Scale bars, 50 μm. Activated caspase 3 positive cells were observed at day 12 and 20 for MCF-10A acini, were not observed for MCF-10AT multi-acinar structures and were observed at days 6, 12, and 20 for MCF-10CA1a masses. The speckled fluorescence outside the acini is from the Matrigel (panel S).

After 6 days in three-dimensional culture MCF-10A acini were negative for activated caspase-3, a marker of apoptosis, but they were positive at days 12 and 20 although there were consistently fewer activated caspase positive cells at day 20 (Table 1, Figure 5A, J, S). Caspase-3 staining was observed in the center of the structures but also in single cells at the outer edge of acini. The MCF-10AT cells, on the other hand, were negative for activated caspase-3 throughout the entire 20-day experiment (Table 1, Figure 5D, M, V). Thus, although MCF-10AT cells formed multiple acinar structures, the lack of activated caspase-3 staining suggests that the programmed cell death that occurs during MCF-10A lumen formation [17] did not occur. In contrast to MCF-10AT, activated caspase-3 staining was observed in proliferating, malignant MCF-10CA1a structures at days 6, 12 and 20 in rBM culture (Table 1, Figure 5G, P, Y). Apoptotic cells were observed both within and at the outer edges of MCF-10CA1a masses.

**Table 1:** Caspase-3 activation observed for MCF-10A, MCF-10AT, and MCF-10CA1a cells grown in three-dimensional culture.

	**Day 6**	**Day 12**	**Day 20**
**MCF-10A**	-	+	+
**MCF-10AT**	-	-	-
**MCF-1-CA1a**	+	+	+

Caspase-3 activation was positive for MCF-10A cells grown in three-dimensional culture at days 12 and 20. MCF-10AT cells were negative for caspase-3 activation throughout the experiment, while MCF-10CA1a cells were positive for caspase-3 activation throughout the experiment.

### Lack of proliferation control in MCF-10AT and MCF-10CA1a cells in three-dimensional cultures

**Figure 6:**
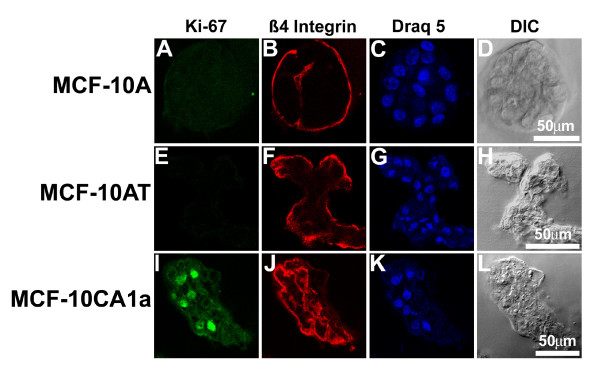
**Ki-67 and β4 integrin staining observed for MCF-10A, MCF-10AT, and MCF-10CA1a cells in three-dimensional culture**. MCF-10A (top), MCF-10AT (middle), and MCF-10CA1a (bottom) cell lines stained to examine proliferation and basement membrane formation during growth in overlay three-dimensional culture. Single confocal sections stained for Ki-67 (A, E, I) indicate that only MCF-10CA1a cells continue to proliferate at day 20 in three-dimensional culture. β4 integrin (B, F, J) staining detects basement membrane deposition for all three cell lines. Nuclei were stained with Draq5 (C, G, K) and matching differential interference contrast images are shown (D, H, L). Scale bars, 50 μm.

**Figure 7:**
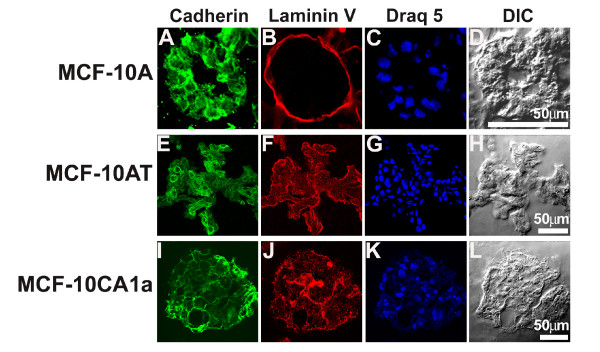
**Cadherin and Laminin V staining observed for MCF-10A, MCF-10AT, and MCF-10CA1a cells in three-dimensional culture**. MCF-10A (top), MCF-10AT (middle), and MCF-10CA1a (bottom) cell lines stained to examine cell:cell junctions and basement membrane formation during growth in overlay three-dimensional culture for 20 days. Single confocal sections of cadherin (A, E, I) demonstrate the existence of cell: cell junctions for all three cell lines at day 20 in three-dimensional culture. Laminin V (B, F, J) staining indicates basement membrane deposition for all three cell lines. Nuclei were stained with Draq5 (C, G, K) and matching differential interference contrast images are shown (D, H, L). Scale bars, 50 μm.

Staining with Ki-67, a marker of cell proliferation [19], indicated that malignant MCF-10CA1a masses continued to proliferate even after 20 days in rBM culture (Figure 6I). By this time, both the MCF-10A and the pre-malignant MCF-10AT cultures had undergone growth arrest and did not have detectable Ki-67 staining (Figure 6A, E). β4 integrin was expressed in structures for all three cell types and was polarized to the outer surfaces of those structures (Figure 6B,F,J). In MCF-10A acini and in MCF-10AT multi-acini, basement membrane formation was complete and limited to the outer edges of the structures (Figure 7B, F). Note the MCF-10A acinus shown in Figure 6B had a notch or invagination with β4 integrin staining, indicative of an irregular surface profile rather than compromised polarity. The basement membrane did not completely surround MCF-10CA1a masses and was also detected at multiple edges within a single mass (Figure 6J).

### Heterogeneous cell: cell junction formation in MCF10-CA1a cells in three-dimensional cultures

Staining of MCF-10A acini for cadherin after 20 days in three-dimensional culture showed clearly defined cell:cell boundaries (Figure 7A). On the same day, MCF-10AT multi-acinar structures also had cadherin at cell:cell boundaries with a more heterogeneous level of expression from cell to cell (Figure 7E). MCF-10CA1a masses had some cadherin at cell:cell boundaries but, unlike structures for the other two cell lines, had high concentrations of cadherin at multiple basal surfaces (Figure 7I). Laminin V expression in MCF-10A cells showed a clearly defined basement membrane surrounding the acinus (Figure 7B), confirming localization of laminin V as reported previously [9]. Similarly, laminin V expression showed a well defined basement membrane surrounding the MCF-10AT multi-acinar structure (Figure 7F), however laminin V expression revealed an incomplete basement membrane with long interior stretches in MCF-10CA1a masses (Figure 7J). This latter observation, along with the decrease in β4 integrin polarity (Figure 6J), suggested that the loss of cell and tissue polarity is a general feature for this fully malignant MCF-10CA1a cell line.

### Altered morphology of MCF-10CA1a cells cultured under different three-dimensional culture conditions

**Figure 8:**
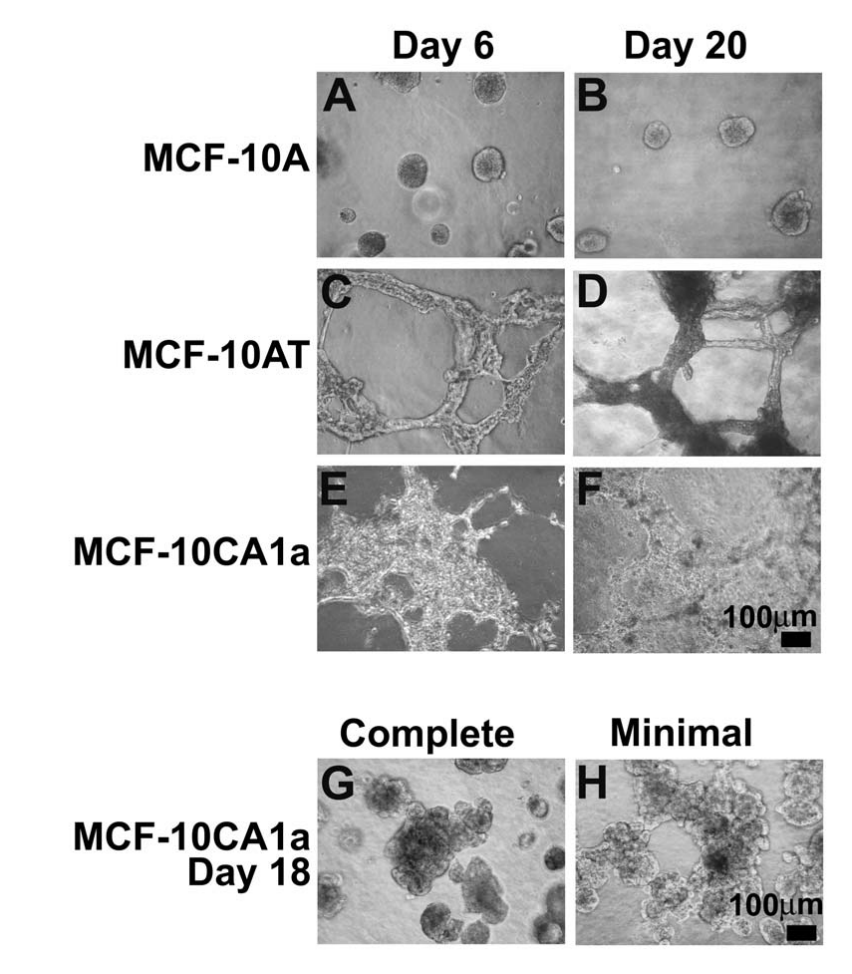
**Morphology observed for MCF-10A, MCF-10AT, and MCF-10CA1a cells grown in three-dimensional culture under varying conditions**. Phase contrast images of MCF-10A (A, B), MCF-10AT (C, D) and MCF-10CA1a (E, F) grown in overlay conditions at a low (0.5%) concentration of Matrigel. MCF-10A cells form spherical acini; MCF-10AT transformed cells form long, branched structures that thicken between days 6 and 20. MCF-10CA1a cells form interconnected masses of cells. Phase contrast images of MCF-10CA1a cells grown in overlay rBM in complete (G) and minimal (H) media. MCF-10CA1a cells at day 18 in 3D culture appear less dense and more spread under the minimal media conditions. Scale bars, 100 μm.

When MCF-10AT and MCF-10CA1a cells were cultured under a lower Matrigel rBM overlay concentration of 0.5% (compared to the usual 2%), highly branched structures with connections between masses, were formed instead of multi-acinar structures or undifferentiated masses (Figure 8C, D, E, F). In contrast, normal MCF-10A cells formed spherical acini under a 0.5% Matrigel overlay (Figure 8A, B) that were similar to those formed with the higher concentration 2% Matrigel overlay (Figure 2). After as early as 2 days in three-dimensional culture under 0.5% Matrigel, both MCF-10AT and MCF-10CA1a cells formed long, thin, branched structures (data not shown). In pre-malignant MCF-10AT three-dimensional cultures these structures thickened and retained distinct edges between days 6 and 20 (Figure 8C, D). Malignant MCF-10CA1a cultures, however, showed a proliferation of cells from the edges of the structures from day 2 to day 6 (data not shown), which continued through day 20. (Figure 8E, F).

Malignant MCF-10CA1a cells can be maintained in monolayer culture in minimal media that lacks hydrocortisone, EGF, insulin, and cholera toxin, whereas the MCF-10A and MCF-10AT cells cannot [15]. When MCF-10CA1a cells were maintained in three-dimensional culture under these minimal media conditions, the resulting cultures displayed large, heterogeneous structures, however, these masses were somewhat more spread and less dense than when the cultures were maintained in complete media (Figure 8G, H).

### Reversion of MCF10AT cells to single acini formation in embedded three-dimensional cultures

**Figure 9:**
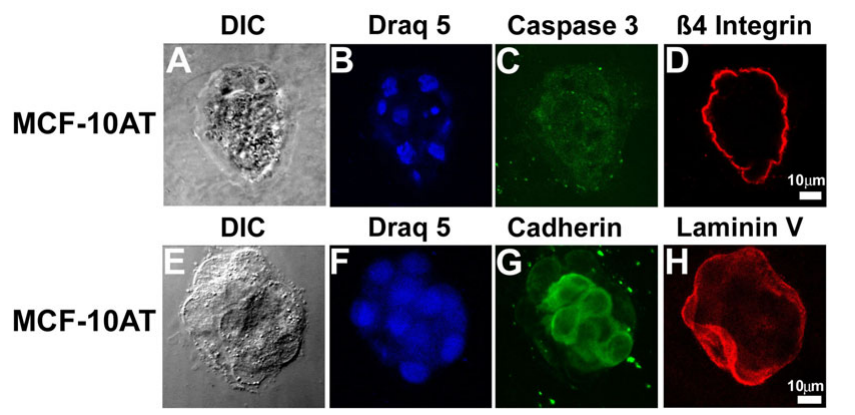
**Altered morphology of MCF-10AT cells in grown for 20 days in embedded three-dimensional culture**. Differential interference contrast (DIC) images (A, E) show that MCF-10AT cells grown under these conditions form irregular acini but not the multi-acinar structures seen in overlay cultures (see Figure 2). Nuclei were stained with Draq5 (B, F). Apoptosis, cell:cell junctions, and basement membrane formation were assessed by confocal microscopy with caspase-3 (C), cadherin (G), β4 integrin (D), and Laminin V (H) staining. Scale bars, 10 μm.

To this point, all observations were made from overlay cultures where cells were plated on a Matrigel layer and overlaid with media containing Matrigel. We subsequently compared characteristics of each cell line when three-dimensional cultures were established by embedding the cells in the lower layer of Matrigel and without including Matrigel in the overlay medium. MCF-10A acini were slightly smaller and more homogeneous in size than when grown in overlay culture, as was previously reported, [17], but otherwise showed no significant difference in apoptosis, basement membrane deposition, or the formation of cell:cell junctions (data not shown). Similarly, the malignant MCF-10CA1a cells showed little difference when the two culture methods were compared (data not shown). However, in embedded culture, MCF-10AT cells formed single acini with irregular edges (Figure 9), but did not form the multi-acinar structures seen in overlay three-dimensional cultures (Figure 2). As previously noted (Figure 5), there was no staining for the apoptosis marker, activated Caspase 3 (Figure 9C), while laminin V containing basement membrane deposition occurred at the basal surface (Figure 9H) with β4 integrin polarized to that surface (Figure 9D). Cadherin labeling indicated that cell:cell junctions formed in the embedded MCF-10AT cultures (Figure 9G), though we noted that cells at the basal surface of these structures consistently lacked cadherin staining.

## Discussion

The MCF-10A progression series grown in three dimensional rBM cultures can be a powerful system for studying human mammary tumorigenesis. All three cell lines derive from a common ancestor and the manipulations that have made them increasingly transformed are well defined. While much can be learned by studying these cells in traditional two dimensional tissue culture, real malignant transformation takes place within a three dimensional tissue. The three dimensional rBM system we have utilized to characterize these cells recapitulates much more of the microenvironment of a tissue and allows for a more detailed investigation of the dynamic and reciprocal crosstalk between the extracellular matrix and nuclear gene expression that may play an important role in real breast tumorigenesis [1,3,5,17,18]. In this study, we assessed cellular parameters of tissue formation in cells of the MCF-10A progression series and documented them as a faithful model of mammary malignant progression.

Variants of differing malignancy have been developed from the first normal human mammary epithelial cell line, HMT-3522 [20], by spontaneous transformation with selection for EGF-independent growth [21-23]). These variants, cultured in rBM, have offered significant insights into tumor progression [24-27]. However, the molecular mechanisms driving these cells toward malignancy were not defined in advance, although some information about the process might be inferred from gene expression studies of the established lines [28].

MCF-10A cells were manipulated by the forced expression of a mutated H-ras-1, which is the human analog of the Harvey sarcoma virus oncogene. This constitutively active Ras variant, which was originally isolated from the bladder carcinoma cell line T24, has a single base mutation causing an amino acid change at residue 12 (Gly to Val) [29]. When injected into nude mice, the resulting MCF-10AT cells persisted as xenografts and ultimately developed into carcinomas 25% of the time [12,13]. In tissue culture studies, expression of this constitutively activated Ras induced transformation of immortalized NIH3T3 cells and of primary rat and hamster cells from multiple tissues, whereas the normal Ras did not [29,30]. While few established breast cancer cell lines have Ras mutations, a majority have Ras overexpression via amplification of the gene [31-33]. More recently, activating mutations in H-ras, K-ras, or N-ras were observed in 8 out of 40 human breast cancer cell lines, with another 4 lines having activating mutations in B-Raf, which is downstream in the Ras pathway [34]. Although only 5% of breast tumors have activating Ras mutations [31], it was shown that more than half had a 2- to 6-fold increase in Ras expression and a more than 5-fold activation in downstream MAP Kinase activity [35]. Thus, Ras signaling may be more commonly activated in breast tumor cells by mechanisms other than mutation. In MCF-10A cells, ErbB1-ErbB2 heterodimerization disrupts acinar tissue structure in three dimensional basement membrane cultures and generates a more invasive phenotype [36]. The invasive phenotype was reverted by inhibition of the Ras/MAP Kinase pathway, supporting a role for the Ras pathway in the malignant progression of ErbB2 expressing tumors. For these reasons, the ectopic expression of activated Ras in MCF-10A cells is likely relevant to the mechanisms of patient breast tumor progression.

While MCF-10AT cells and normal MCF-10A cells grown in three-dimensional reconstituted basement membrane culture both exhibited growth arrest, this arrest appeared to be delayed in the MCF-10AT cells. These cells also demonstrated a nearly complete lack of apoptosis, resulting in the absence of notable lumen formation. A recently published report indicated that in MCF-10AT cells grown in three-dimensional culture, apoptosis was decreased, but not absent, leading to reduced lumen formation [37]. MCF-10AT cells were normal or nearly normal in their ability to deposit basement membrane and to form cell:cell junctions. Thus the activation of Ras predominantly affects the proliferation state of the cells in three-dimensional culture, consistent with the idea that these cells recapitulate aspects of breast tissue hyperplasia *in vivo *[38].

In contrast, the malignant MCF-10CA1a cells never exhibited growth arrest and never formed normal acinar structures, consistent with reports comparing primary mammary epithelial cells, primary breast tumor cells, and transformed breast cancer cell lines [4]. The MCF-10CA1a cells exhibited unchecked proliferation in three-dimensional culture despite the continuous occurrence of apoptosis, suggesting the loss of normal growth controls. In addition, the MCF-10CA1a cells showed an incomplete deposition of basement membrane proteins and abnormal cell:cell contacts. Interestingly, the expression levels of the markers assayed in these experiments were not significantly altered. Instead, the proper structural organization of the proteins within the cells was compromised. These phenotypes are consistent with more advanced stages of tumorigenesis, at which the dynamic relationships between cell:cell interaction and cell:ECM contact are altered [1,39].

These progressively malignant cell lines were engineered in the same cell background by a defined series of manipulations. They recapitulate tumor phenotypes in a three dimensional culture system, and this will facilitate future studies of malignant progression. Compared to *in vivo *studies, organotypic culture systems are easier and less expensive to manipulate, facilitate fluorescence based microscopy imaging, have a reduced complexity of cell types in a simplified microenvironment, and are influenced by a reduced number of uncontrolled factors. This simplicity can be a disadvantage, ignoring features not included in the tissue culture, but it can also be a great advantage for mechanistic studies of malignant changes. Of particular interest is the potential use of these cell lines in three dimensional culture to dissect the signal transduction pathways, and especially the ras pathway, that drive these cells to increasing malignancy.

## Conclusion

Cancer arises from a complex interaction of factors including both genetic changes as well as changes in the microenvironment [40,41]. We have combined the isogenic MCF-10A progression series of cell lines, where cells have been pushed toward malignancy by a defined molecular manipulation, with a three dimensional tissue culture system that has already provided powerful insights into breast tissue morphogenesis and tumorigenesis. The results show that this progression series can be an excellent model system for the better understanding of breast cancer.

## Methods

### Cell Culture

MCF-10A, MCF-10AT (formally known as MCF-10AneoT), and MCF-10CA1a cells were obtained from the Barbara Ann Karmanos Cancer Institute (Detroit, MI). The three cell lines were maintained in monolayer in Dulbecco's modified Eagle's medium-F12 (DMEM/F12) (Invitrogen, 21041025) supplemented with 5% horse serum (Invitrogen, 16050122), 1% penicillin/streptomycin (Invitrogen, 15140122), 0.5 μg/ml hydrocortisone (Sigma, H-0888), 100 ng/ml cholera toxin (Sigma, C-8052), 10 μg/ml insulin (Sigma, I-1882), and 20 ng/ml recombinant human EGF (Peprotech, 100-15).

### Three-dimensional Cell Culture

MCF-10A, MCF-10AT, and MCF-10CA1a cells were cultured on plastic tissue culture dishes (Nalge Nunc, Rochester, NY,172958) in either Reduced Growth Factor Matrigel without phenol red or Non-Reduced Growth Factor Matrigel with phenol red (BD Biosciences, San Jose, CA, #3562312 and #356234) following either the overlay or embedded procedures of Debnath et. al. [9]. Briefly, for overlay cultures, cells were prepared for three-dimensional culture by growing to 20–80% confluency in monolayer and seeding in a single cell suspension on 100 μl of Matrigel in a 35mm plate at 7,000 to 15,000 cells/plate or on 40 μl Matrigel in a 4 well chamber slide (Nunc, Lab-Tek, 177399) at 5000 cells/well. For embedded cultures, cells were grown up to 80% confluence in monolayer culture and plated at 2.5 × 10^4 ^cells/ml in 300 μl Matrigel, on top of a thin layer of 75 μl Matrigel, in each well of a 4 well chamber slide (see Figure 8A). In addition, some overlay three-dimensional cultures of MCF-10CA1a cells were prepared with cells grown in monolayer under minimal media conditions, consisting of DMEM/F12 with 5% horse serum, 0.029M sodium bicarbonate, 10mM HEPES and 1% penicillin/streptomycin (see Figure 8, panels G and H).

Once plated on rBM, all cultures were incubated at 37°C in a 5% CO_2 _humidified incubator for up to 20 days. Three-dimensional cultures were grown in assay media [9] consisting of DMEM/F12 containing 2% horse serum, 5 ng/ml EGF, and 2% Matrigel (for overlay cultures only). Media was replaced every 2 or 4 days. Morphology was observed every 2 days via phase contrast microscopy. Size of resulting structures were measured every 2 days and analyzed via an analysis of variance (ANOVA) test followed by a Tukey adjustment.

### Cryoblock Preparation

MCF-10A, MCF-10AT, and MCF-10CA1a cells were harvested at day 6, 12 or 20 by first washing with warm assay media, followed by fixing with 4% formaldehyde in PBS for 20 minutes at room temperature, then permeabilizing with 0.5% Triton X-100 in PBS for 10 minutes at 4°C. Cultures were rinsed with a glycine rinse (130 mM NaCl, 7 mM Na_2_HPO_4_, 3.5 mM NaH_2_PO_4_, 100 mM glycine) three times for 20 minutes each at room temperature, followed by a blocking step with TBS-1 (10 mM Tris HCL, pH 7.7, 150 mM NaCl, 3 mM KCl, 1.5 mM MgCl_2_, 0.05% (v/v) Tween 20, 0.1% (w/v) bovine serum albumin, 0.2% (w/v) glycine) for 30 to 40 minutes at room temperature. Cultures were transferred to 1.5 ml Eppendorf tubes; TBS-1 was aspirated and replaced with 15% sucrose for 15 minutes at room temperature, aspirated, and replaced with 30% sucrose for 15 minutes at room temperature. Sucrose was replaced with Tissue Tek O.T.C. compound (Sakura Finetek, #4583, Torrance, CA) or with TBS Tissue freezing medium (Triangle Biomedical Sciences, H-TFM, Durham, NC) and samples were flash frozen in liquid nitrogen and stored at -80°C.

### Immunofluorescence

Antibodies used for immunostaining included β4 integrin (Chemicon, MAB#1964), human Laminin V (Chemicon, MAB#19562), pan-Cadherin(Sigma, #C3678), Ki-67 (Zymed, #18-0191), and cleaved Caspase 3 (Cell Signaling, #9664).

To immunostain three-dimensional samples, a thin layer of Matrigel (10 μl) was used to coat the bottom of each well of a 4-well slide and allowed to gel for at least 20 minutes at 37°C. A 10 or 20 μl sample of cells was layered on top of the thin Matrigel layer and incubated for at least 20 minutes at 37°C. Samples were then washed with warm media, followed by one of three fixing methods. Cells were either fixed and then permeabilized or permeabilized and then fixed using 4% formaldehyde in PBS for 20 minutes at room temperature as the fixative, and using 0.5% Triton X-100 in PBS, 10 minutes at 4°C for permeabilization. Alternatively, for caspase staining (Figure 5) cultures were fixed with methanol: acetone (1:1) for 20 minutes at -20°C. Following all fixation methods, samples were rinsed with a glycine rinse, 3 times for at least 10 minutes each time while rocking gently. This step was followed by a block with IF buffer (130 mM NaCl, 7 mM Na_2_HPO_4_, 3.5 mM NaH_2_PO_4_, 7.7 mM NaN_3_, 0.1% Bovine Serum Albumin, 0.2% Triton X-100, 0.05% Tween 20) containing 10% goat serum (Invitrogen, #10000C) for 1 hour 30 minutes, followed by a secondary block in IF buffer containing 10% goat serum and 20 μg/ml F(ab')_2 _(Jackson Laboratories, #115-006-006) for 30 to 40 minutes. Samples were incubated with primary antibodies in secondary block solution overnight at 4°C. Antibody concentrations used were β4 integrin (1:20), Laminin V and pan-cadherin (1:200), Ki-67 and estrogen receptor (1:100), and caspase (1:200). Samples were washed three times for 20 minutes in IF buffer while gently rocking, followed by a 1-hour incubation with the appropriate Alexa-conjugated secondary antibodies (Molecular Probes) at a concentration of 1:200. Nuclei were counterstained with Draq 5 (Alexis Biochemicals, BOS-889-001-R200) prior to a final wash with PBS and mounting with Prolong Antifade Kit (Molecular Probes, P-7481, Eugene OR).

To immunostain cryo-sections, they were first allowed to warm to room temperature in a humidity chamber for 15 minutes. All subsequent steps, except primary antibody incubation, were carried out at room temperature. The sections were washed with PBS with 0.05% Tween 20 for 20 minutes. Sections were blocked with IF buffer containing 10% goat serum for 1 hour 30 minutes, followed by a second block in IF buffer, 10% goat serum and 20 μg/ml F(ab')_2 _for 30 to 40 minutes. Sections were washed for 20 minutes in Signal Enhancer (Image-iT FX, Invitrogen, #136933) and incubated with primary antibodies in IF buffer with 10% goat serum overnight at 4°C. Antibody concentrations were as described above. Sections were washed three times for 20 minutes each in IF buffer followed by 1 hour incubation with appropriate secondary antibodies (Molecular Probes) at a concentration of 1:2000. Sections were again rinsed 3 times with IF buffer for 20 minutes each followed by labeling with Draq 5, and a final wash with PBS before mounting with Prolong Antifade Kit. Images were captured using laser scanning confocal microscopy with a Leica SP1.

## Abbreviations

rBM: reduced basement membrane; DMEM/F12: Dulbecco's modified Eagle's medium-F12; PBS: phosphate-buffered saline; IF: immuno-fluorescence.

## Competing interests

The authors declare that they have no competing interests.

## Authors' contributions

KI designed and carried out all of the experiments, and prepared the manuscript. IT carried out some of the experiments. AI and JN conceived and designed experiments and critically reviewed the manuscript.
